# Does brain entrainment using binaural auditory beats affect pain perception in acute and chronic pain?: a systematic review

**DOI:** 10.1186/s12906-024-04339-y

**Published:** 2024-01-12

**Authors:** Fatemeh Shamsi, Fatemeh Azadinia, Maryam Shaygan

**Affiliations:** 1 Community Based Psychiatric Care Research Center, School of Nursing and Midwifery, Shiraz University of Medical Sciences, PO Box 71345-1359, Shiraz, Iran; 2 Rehabilitation Research Center, Department of Orthotics and Prosthetics, School of Rehabilitation Sciences, Iran University of Medical Sciences, Tehran, Iran

**Keywords:** Binaural beats, Pain, Analgesia, Hemispheric synchronization

## Abstract

**Background:**

Pain is a major clinical problem across all ages with serious social and economic consequences and a great negative impact on quality of life. Brain entrainment using binaural beats is a non-pharmaceutical intervention that is claimed to have analgesic effects in acute and chronic pain. We aimed to systematically review the available randomized clinical trials on the efficacy of binaural auditory beats in reducing adults’ pain perception in acute and chronic pain. A systematic search in electronic databases including Medline (via PubMed), Web of Science, Scopus, Cochrane Central Register of Controlled Trials (CENTRAL), and Embase was performed. The search was completed through Google Scholar and a manual search of the reference lists of all included studies. Randomized clinical trials with full text available in English that investigated the effect of binaural auditory beats on pain perception in acute and chronic pain in adults were included. The risk of bias was assessed by the revised Cochrane risk-of-bias (RoB 2) tool. Furthermore, The GRADE (Grading of Recommendations Assessment, Development and Evaluation) approach was used to assess the quality of the evidence. Sixteen studies (three on chronic pain and thirteen on acute pain perception) fulfilled the eligibility criteria. Because of substantial heterogeneity of the studies, a meta-analysis was inappropriate and this review focused on the narrative interpretation of the results. The risk of bias in most studies was high and the quality of evidence was low to very low. Although the effects of binaural beats on pain perception seem to be influenced by the etiology of pain or medical procedures, our review identifies alpha or a combination of tones in the range of delta to alpha as a potential non-pharmacological intervention in reducing acute pain. However, drawing a conclusion regarding the efficacy of binaural beats for chronic pain requires more high-quality studies.

**Registration:**

The protocol of this review was registered in PROSPERO (No. CRD42023425091).

**Supplementary Information:**

The online version contains supplementary material available at 10.1186/s12906-024-04339-y.

## Introduction

Pain is a major clinical problem across all ages with serious social and economic consequences. In addition, pain conditions have a great negative impact on quality of life and contribute highly to disability around the world [1, 2]. The revised definition of pain offered by the International Association for the Study of Pain in 2020 describes pain as an undesirable experience having sensory and emotional dimensions that is associated with or seems to be associated with, actual or potential tissue injury [3].

Pain perception is highly subjective. The physiological, emotional, and cognitive states of the individuals can influence levels of perceived pain so that an individual may experience different levels of pain in various contexts even when there is no change in the level of noxious stimulus [4, 5]. The experience of pain, as an integrative phenomenon resulting from dynamic interactions of diverse sensory and contextual processes, is associated with brain oscillations at different frequencies [6]. Previous studies have revealed that noxious stimuli induce alterations in particular brain activity rhythms [7, 8]. Increased neural activity at low frequencies (below 10 Hz) [9] and suppression at alpha and beta frequencies [10], as well as induced gamma oscillations at milliseconds after applying a painful stimulus, have been reported [11]. In addition, theta and beta overactivations have been noticed in patients with chronic pain [12, 13]. Thus, different brain stimulation techniques that can modulate these responses have been used to relieve pain in different conditions [14, 15].

Brain entrainment using binaural beats is a non-pharmaceutical intervention that is claimed to affect cognition and psychophysiological states [16]. When two sinusoidal tones with different frequencies are presented simultaneously and independently to each ear, a single illusionary tone called a binaural beat, is perceived by the subject that its frequency equals the difference between the two inputs [17]. For instance, presenting a tone of 400 Hz to one ear and a tone of 412 Hz to the other will result in a perceived tone that fluctuates in amplitude with a frequency of 12 Hz [18]. A change in the relative power of electro-cortical activity of the brain and its synchronization with the frequency of the externally presented stimulus, referred to as the frequency following response, has been suggested as the underlying mechanism of brain entrainment through binaural beats [19].

The brain’s electrical response to pain has been targeted by binaural auditory beats stimulation to induce analgesic effects in both acute [20] and chronic pain [21] in previous researches. Some studies have reported reduced analgesic requirements during surgery [22, 23] or lower perceived acute pain during medical procedures such as colonoscopy [24] and cystoscopy [25] following binaural auditory beats stimulation. However, Roshani et al. (2019) did not find an effect of binaural beats on the level of pain perceived by patients under eye surgery compared to conventional treatment [26]. Regarding chronic pain, a few studies that applied binaural beats for chronic pain have reported different results. Zampi [21] and Gkolias et al. [27] reported reduced pain following binaural beats intervention compared with sham stimulation, while Thanyawinichkul et al. [28] did not find intergroup differences between binaural beats and sham stimulation in people suffering from chronic pain. Two meta-analyses have reported the efficacy of binaural beats on pain perception [16, 29]. In a meta-analysis that aimed to assess the effects of binaural beats on acute pain, Garcia-Argibay et al. found a medium, notable effect for binaural auditory beats in reducing pain perception during surgery [16]. Furthermore, results of another meta-analysis by Maddison et al. considering sensory stimulation in both visual and auditory forms, suggest that neural auditory entrainment can alleviate acute and chronic pain [29]. However, Garcia-Argibay et al. included only three articles recruiting patients under surgery [16], and the review by Maddison et al. was restricted to seven studies that considered binaural auditory beats, including five studies on acute pain in different medical procedures and two on chronic pain [29]. The growing interest in using binaural auditory beats for pain management in recent years has resulted in newly published studies with controversial results [28, 30]. According to this growing attention and controversial findings, our study aimed to systematically review the available randomized clinical trials to determine whether binaural auditory beats can influence adults’ pain perception in both acute and chronic pain.

## Materials and methods

This review complies with the Preferred Reporting Items for Systematic Reviews and Meta-Analyses (PRISMA) guidelines (S1 Table) [31]. The protocol of this review was also registered in the International Prospective Register of Systematic Reviews (PROSPERO) (registration No. CRD42023425091).

### Information sources

A systematic search in electronic databases, including Medline (via PubMed), Web of Science, Scopus, Cochrane Central Register of Controlled Trials (CENTRAL), and Embase was performed. In order to identify additional eligible studies, the literature search was completed through Google Scholar, and the references of all included studies were also manually checked.

### Search strategy

A combination of keywords, defined based on the inclusion criteria of the study, was used to find relevant studies from inception to April 2023. To find all related studies, no limitation was applied with regard to clinical conditions, participants, and publication date.

The search details in PubMed were as follows:

**((pain[Title/Abstract] OR pain[MeSH Terms] OR ache[Title/Abstract] OR ache[MeSH Terms] OR analgesia[Title/Abstract] OR analgesia[MeSH Terms]) AND (binaural beat[Title/Abstract] OR binaural beats[Title/Abstract] OR binaural auditory beat[Title/Abstract] OR binaural beat entrainment[Title/Abstract] OR hemispheric synchronization[Title/Abstract])).**

The basic search was appropriately changed to optimize the strategy for other databases (S1 File). The reference management software EndNote V.X9 (Clarivate Analytics) was used for data management.

### Inclusion and exclusion criteria

After removing all duplicate articles, two independent reviewers (FSH and FA) screened the titles and abstracts of the remaining records to identify relevant papers based on the inclusion-exclusion criteria. If sufficient data were not provided in the abstract for inclusion, the full text was considered. Any disagreement regarding including an article was discussed until a consensus was reached.

The following criteria were considered to include studies in the final list for review:

1. Studies in the English language that were published in peer-reviewed journals and their full texts were available. Conference proceedings and results obtained from a thesis were excluded.
2. Randomized clinical trials in which binaural auditory beats stimulation was used as the main intervention. Nonrandomized experimental studies, feasibility studies, and case reports were excluded.
3. Experimental or clinical studies that recruited human adults older than 18 years old with acute or chronic pain.
4. Studies that reported pain scores or analgesic consumption as a measure of pain perception in acute or chronic pain.

### Data extraction

Data extraction from the included studies for descriptive analyses was done independently by two reviewers (FSH and FA). If there was any disagreement between the reviewers, it was discussed until a consensus was reached. The extracted information for each study included the first author’s name and publication year, study design, characteristics of participants (sex, age, state of health), the number of participants, intervention details (frequency of binaural beats, moment and duration of exposure), control/comparison group, outcome measures, and findings. For trials with more than two arms, the data were extracted for the binaural beats and control arms.

### Evaluating the risk of bias

The revised Cochrane risk-of-bias tool (RoB2) was used to evaluate the risk of bias for each included study [32]. This tool addresses biases categorized into five domains arising from 1) the randomization method; (2) deviations from predesignated interventions; (3) the absence of outcome data; (4) outcome measurement; and (5) selective reporting of findings. Each domain is judged as “low risk of bias,” “some concerns,” or “high risk of bias” [32]. Two reviewers did the risk of bias assessment independently. Any disagreement was resolved by discussion.

### Quality of evidence assessment

The quality of the evidence was assessed for the main outcomes using the Grading of Recommendations Assessment, Development, and Evaluation (GRADE) approach. Two reviewers graded the level of evidence independently. Five factors, including limitations, inconsistency, indirectness, imprecision, and publication bias were considered for rating the quality of evidence as high, moderate, low, and very low. Chronic pain perception, acute pain perception, and analgesic consumption were relevant outcomes for quality assessment [33].

### Summary measures and data synthesis

Given the substantial heterogeneity of the studies (i.e., medical procedures and interventional settings, such as duration and time of binaural beats exposure, frequency of binaural beats, pain etiology, etc.), a meta-analysis is inappropriate. Therefore, the focus of this review is on the narrative interpretation of the results. The included studies were categorized according to outcome measures into three groups, including chronic pain perception, acute pain perception, and analgesic consumption. To visualize quantitative data, we provided forest plots, which represent effect estimates and their confidence interval for each study without producing the overall estimate of effect. Results were reported using mean and standard deviation (SD). When confidence intervals were reported, SD was calculated using the formula: *SD* = √N *(CI-upper limit- CI-lower limit)/3.92; N: sample size, CI: confidence interval [34]. Differences between the binaural beats and control groups were summarized using the standardized mean difference (SMD) and 95% confidence intervals (95% CI).

RevMan software (v.5.4 Cochrane Collaboration) was used for producing forest plots [35].

## Results

**Fig. 1:**
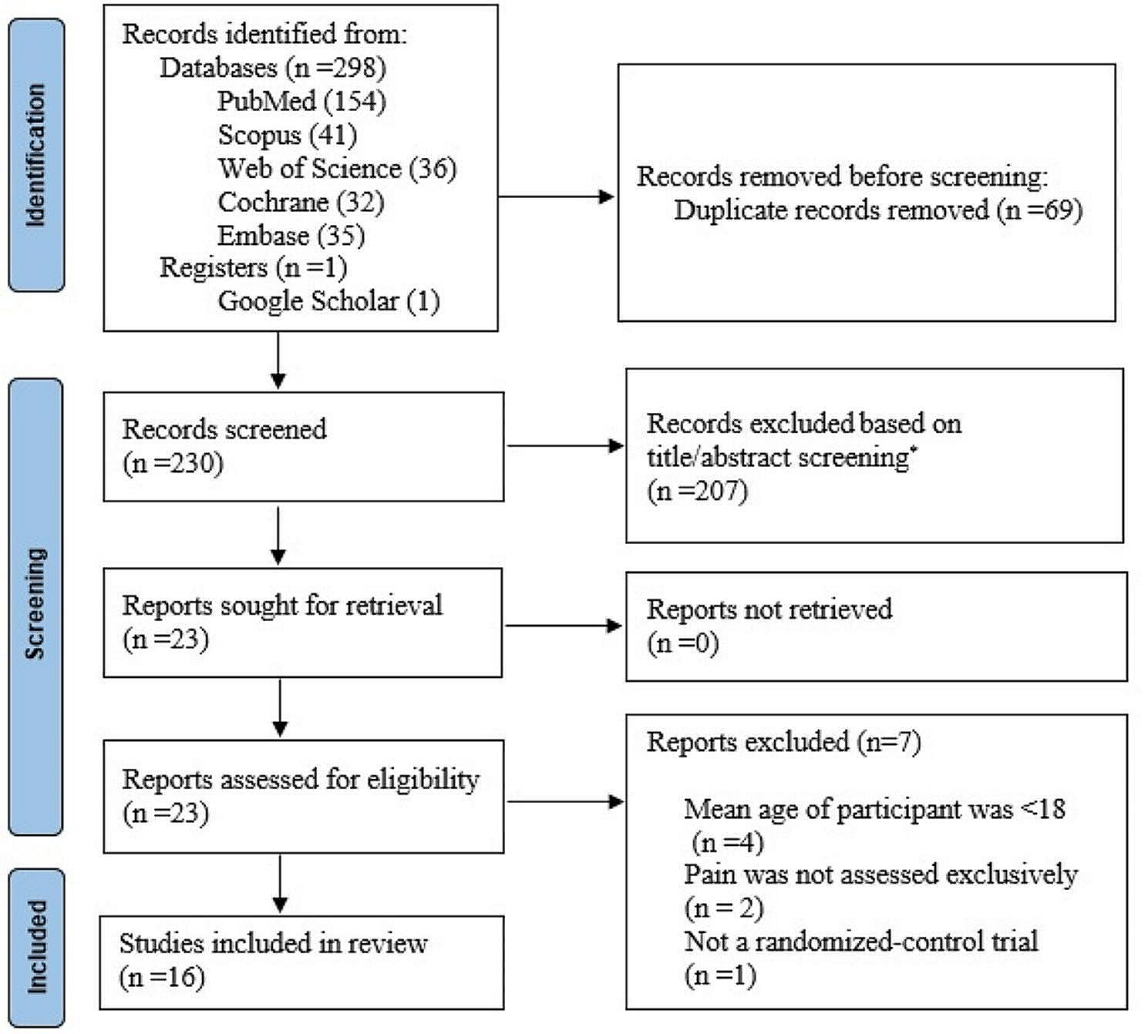
The PRISMA 2020 flow diagram for the search strategy and study selection. * Records that did not meet the inclusion criteria based on title/abstract screening were excluded

A total of 298 references were retrieved from 5 databases. Searching Google Scholar yielded one additional study. Sixty-nine duplicates were identified by EndNote and removed. Then, titles and abstracts of the remaining 230 articles were reviewed which resulted in the exclusion of 207 articles. Full texts of 23 articles were downloaded and assessed for eligibility [20–28, 30, 36–48] from which 7 articles were excluded because they recruited participants younger than 18 years (4 articles) [41, 46–48], had no pain assessment (2 articles) [23, 39], and were not an RCTs (1 article) [44]. Finally, 16 studies [20–22, 24–28, 30, 36–38, 40, 42, 43, 45] were included in the systematic review among which seven studies [20–22, 25, 27, 36, 37] overlapped with previous meta-analyses [16, 29]. The process of study selection is illustrated in detail in Fig. 1.

### Characteristics of included studies

Sixteen studies were included in this systematic review. Characteristics of the included studies are summarized in Table 1.

**Table 1:** Characteristics of 16 included studies

Author (year)	Study design	Participants demographics				Intervention description		Comparison group	Outcome measures	Results
		Sex	age Mean/ median (SD/range)	Health state	N	Type of intervention/frequency of the tone	Moment /duration of exposure			
Bae et al. (2023) (42)	Single-blind, 3-arm, parallel-group	M/F BB = 15/49 C = 14/46	BB = 67 (64–72) C = 65 (58–70)	Adult underwent sedation with dexmedetomidine during spinal anesthesia for elective surgery	BB = 63 C = 60	Real-time binaural modulated music/ frequencies of 8, 6, and 3 Hz	During surgery/ duration of surgery BB = 80 [68–100]^a^ C = 88 [70–110]	Blank tape	Dexmedetomidine loading dose/PBW, dexmedetomidine loading dose (μg), dexmedetomidine total dose (μg), and total loading time	Real-time binaural music reduced the loading dose of dexmedetomidine for adequate sedation
Nelson et al. (2023) (44)	Non-blind,3-arm, parallel-group	Female	Mean age not reported (Over 40 y/o)	Patients who attended for mammography	BB = 20 C = 20	Binaural beat/ NR	Before procedure/ 5 min	Standard care	Pain using a validated numerical rating scale immediately after the compression was released	5 min exposure prior to mammographic compression gave an improvement in the perception of patient pain from reported pain scales in the BB group
Loong et al. (2022) (41)	Double-blind, 2-arm, parallel-group	M/F BB = 14/17 C = 19/11	BB = 67.7 (9) C = 63.9 (6.2)	Subjects with senile cataracts undergoing phacoemulsification under topical anesthesia	BB = 31 C = 30	Binaural beats/ 10 Hz	Before surgery/ 10 min	Blank tape	The Patient’s pain scores using VAS immediately after the surgery	The binaural beat group had significantly lower pain scores than the control group
Tani et al. (2022) (24)	Double-blind, 2-arm, parallel-group	M/F BB = 24/18 C = 26/22	BB = 58.8 (12.83) C = 61.6 (9.72)	Patients underwent colonoscopy examination	BB = 42 C = 48	Binaural beat with a white noise background/ 4 Hz	Five min before and during the procedure/ duration not reported	Background white noise alone	Level of the feeling of pain during the procedure using VAS	The BB group perceived a lower feeling of pain than the control group
Thanyawinichkul et al. (2022) (28)	Double-blind, 2-arm, parallel-group	M/F BB = 4/6 C = 2/10	BB = 51.3 (16.34) C = 53.5 (12.99)	Patients with moderate to severe chronic back pain	BB = 10 C = 12	Binaural beat combined with acoustic piano music/ 6 Hz	20-minutes per day for 14 days	Acoustic piano music with a frequency of 300 Hz.	Measurement of two aspects of pain including pain severity and pain interference comprised of: - Body pain mapping - Pain intensity using Thai BPI	No intergroup differences were found in any of the outcomes
Olcucu et al(2021) (25)	Non-blind,3-arm, parallel-group	Male	BB = 56.26 (14.93) C = 55.56 (16.41)	Patients undergoing cystoscopy	BB = 61 C = 75	Binaural beats without background/ 10 Hz	10 min before and during procedures/ duration of procedures BB = 6.31 (1.08)^b^ C = 5.97 (0.9)	Blank tape	The Patient’s pain scores using VAS	Significantly lower VAS scores in the BB group compared to the control after the procedure
	Non-blind,3-arm, parallel-group	Male	BB = 41.95 (14.54) C = 48.53 (14.19)	Patients undergoing ureteral stent removal	BB = 41 C = 52	Binaural beats without background/ 10 Hz	10 min before and during procedures/ duration of procedures BB = 2.92 (0.52)^b^ C = 2.84 (0.63)	Blank tape	The Patient’s pain scores using VAS	Significantly lower VAS scores in the BB group compared to the control after the procedure
Tani et al. (2021) (30)	Double-blind, 2-arm, parallel-group	M/F BB = 6/14 C = 7/13	BB = 75.65 (5.2) C = 73.65 (6)	Patients underwent total knee joint replacement with spinal anesthesia	BB = 20 C = 20	Binaural beats/ 4 Hz	Before surgery/ 20 min	Acoustical stimulation at 256 Hz	The amount of morphine consumption displayed on the PCA device and the NRS values fat 8, 16, and 24 h after surgery	PCA morphine consumption in the intervention group after surgery was significantly lower than that of the control group, No significant difference in pain perception after surgery
Gkolias et al. (2020) (27)	Double-blind, 2-arm, cross-over	48% male	58.76 (14.63)	Patients with chronic pain (pain due to cervical or lumbar spine disorders, neuralgia, rheumatic disease, or other diseases )	BB = 20 C = 20	Binaural beat with soft music in the background/ 5 Hz	30 min for a single-session study, using on-demand during a week for long-term effect	Soft music at 400 Hz	Self-reported pain in NRS at baseline and both after the brief 30-minute intervention and at the end of the intervention week, everyday mean levels of pain and analgesic medication during baseline and the intervention week	Theta rhythm binaural beat application significantly reduced pain intensity and everyday analgesic medication use, compared to sham intervention
Roshani et al. (2019) (26)	Non-blind,2-arm, parallel-group	M/F BB = 16/14 C = 13/17	BB = 57.46 (4.26) C = 57.56 (6.03)	Patients underwent eye surgery with anesthesia	BB = 30 C = 30	Binaural beat/ NR	5 min before and during surgery/ duration of surgery not reported	conventional treatment	The patient’s severity of pain assessed by VAS	The decreased pain level in the BB group after surgery, while pain scores showed no between-group difference before and after surgery
Kurdi and Gasti (2018) (39)	Non-blind,3-arm, parallel-group	Female	BB = 24.6 (2.9) C = 24.5 (2.7)	Parturient underwent emergency cesarean section delivery under spinal anesthesia	BB = 59 C = 62	Binaural beat/ NR	During the surgery/ duration not reported	Blank tape	The intensity of pain using VAS at 1, 6, and 24 h after surgery and the mean time required for the first rescue analgesic	Mean pain scores at 6 and 24 h post-surgery were significantly lower in the BB group, a significant between-group difference at the mean time required for the 1st rescue analgesia
Ecsy et al. (2017) (20)	Blinded, 2-arm, cross over	M/F = 16/16	23.25 (7.9)	Healthy	BB = 32 C = 32	Binaural beat/ 8,10, and 12 Hz	10 min for each frequency, a total of 30 min	10 min of white noise, 3 times, a total of 30 min	Perception of acute pain using rating the pain induced by 30 painful heat laser pulses	Pain ratings after all alpha frequency entrainment were all significantly different from all three control conditions with the largest after 10 Hz stimulation
Zampi (2015) (21)	Single-blind ,2-arm, cross over	M/F= 19%/81%	47 (26–69)	Chronic pain based on patients’ self-report ( headaches, back and lower-back pain, fibromyalgia, lower-spinal birth defects, sciatica, myofascial pain, neck/ knee/ hip pain, joint aches)	BB = 32 C = 32	Binaural beat/ 6-Hz	20 min daily, 14 consecutive days for each situation	A single tone at 300 Hz	Haven-Yale MPI’s average score on the subscale for pain severity pre-test, and the average of 2 post-test scores	A significant main effect on the change in perceived pain severity for the binaural beat group
Bălan (2014) (37)	Non-blind,3-arm, parallel-group	M/F = 20/27	BB = 43 (7.01) C = 30.68 (2.12)	Healthy	BB = 16 C = 15	Binaural beat / combination of tones within delta to the alpha range	5 min	No intervention	The level of perceived discomfort caused by a painful stimulus during each situation using a 10-point Likert	The participants exposed to binaural beats, reported lower levels of perceived pain, compared to those who did not receive any treatment
Dabu-Bondoc et al. (2010) (36)	Double-blind, 3-arm, parallel-group	M/F% H = 38/62 C = 28/72	H = 42 (14) C = 41(10)	Outpatients underwent surgery requiring general anesthesia	H = 20 C = 20	Binaural beat (Hemi-sync)/ NR	30 min Before surgery and during surgery /duration of surgery not mentioned	Blank cassette tape producing white noise	Intraoperative fentanyl, Perioperative analgesic consumption at PACU and home, measurement of pain scores using VAS at 10, 20,30, 60 min, and 24 h post operation	The Hemi-sync group required significantly less fentanyl during the anesthetic procedure compared with control group. Pain VAS scores at 1 h in the PACU and at 24 h after surgery were significantly lower in the treatment group compared with and control.
Lewis et al. (2004) (35)	Double-blind, 2-arm, parallel-group	NR	H = 56 (16) C = 52 (11)	Patients underwent lumbar spine surgical procedures	H = 15 C = 15	Binaural beat (Hemi-sync)/ NR	Before and during surgery/ duration of surgery H = 170 (61)^b^ C = 176 (73)	Blank tape	Fentanyl administration during operation	No significant difference in fentanyl requirements between
	Double-blind, 2-arm, parallel-group	NR	H = 38 (10) C = 41 (10)	Patients underwent bariatric surgical procedures	H = 15 C = 15	Binaural beat (Hemi-sync)/ NR	Before and during surgery/ duration of surgery H = 130 (74)^b^ C = 136 (26)	Blank tape	Fentanyl administration during operation	Bariatric patients who listened to Hemi-Sync received less fentanyl per kilogram per minute
Kliempt et al.(1999) (22)	Double-blind, 3-arm, parallel-group	M/F H = 15/10 C = 9/17	18–76 F/m H = 49 (12.6)/37 (15.6 ) C = 49 (11.5)4/ 3(19.9)	Patients underwent general surgical operations requiring general anesthesia	H = 25 C = 26	Binaural beat (Hemi-sync)/ NR	During surgery/ duration of surgery H = 63 (41)^b^ C = 48 (28)	Blank tape	Fentanyl administration during operation	Patients in the Hemi-Sync group required less fentanyl compared with the blank tape group

SD, standard deviation; N, Number of participants; M, male; F, female; BB, binaural beat; C, control group; PBW, predicted body weigh; NR, not reported; VAS, visual analog scale; BPI, Brief Pain Inventory; ; PCA, Patient-controlled analgesia; NRS, numeric rating scale; MPI, Multidimensional Pain Inventory; Hemi-sync, Hemispheric-synchronized sounds; PACU, post-anesthesia care unit

^a^ Mean [range] in minutes; ^b^ mean (standard deviation) in minutes

Of 16 included studies, 3 used binaural beats intervention in chronic pain [21, 27, 28], and 13 remaining studies assessed the effect of binaural beats on acute pain perception either in patients undergoing a medical procedure [22, 24–26, 30, 36, 37, 40, 42, 43, 45] or healthy participants in an experimental situation [20, 38].

A substantial heterogeneity was found in the included studies with respect to the binaural beats exposure time and duration, the frequency of applied binaural beats, comparison group, and patients’ medical conditions so that they included patients undergone different surgeries or medical procedures. Therefore, meta-analysis was inappropriate and quantitative results in each individual study are presented in forest plots produced by RevMan., The reported effect sizes based on mean difference are interpreted as small (0.0–0.2), medium (0.4–0.5), and large (0.8–3.0) effects [49].

### Chronic pain

#### Participants’ characteristics and study setting

The number of subjects recruited in studies on chronic pain ranged from 10 to 32 in each study arm. All three studies recruited both genders (male and female) with ages ranging from 26 to 69 years old [21, 27, 28]. Patients in the two studies suffered chronic pain with different etiologies, including musculoskeletal disorders, neuralgia, fibromyalgia, rheumatic disease, etc. [21, 27]. , while the patients in the study by Thanyawinichkul et al. (2022) had chronic back pain [28].

#### Intervention features

The binaural beats exposure time was 280 min in two studies (20 min a day for 14 days) [21, 28], while in one study, the maximum time of listening to binaural beats was dependent on participants’ demand during a one-week intervention with a minimum of 30 min exposure in a single-session study design [27]. All three studies used binaural beats in the theta frequency range (6 Hz in two studies and 5 Hz in one), combined with music [27, 28] or alone [21], compared with a sham situation of listening to a single tone at 300 [21, 28] or 400 Hz [27].

#### Measurements

Each study used a different scale to measure pain, including the Haven-Yale scale, Multidimensional Pain Inventory (MPI) [21], numeric rating scale [27], and Thai Brief Pain Inventory [28].

### Acute pain

#### Participants’ characteristics and study setting

The number of subjects recruited in studies on acute pain ranged from 15 to 84 in each study arm. Nine out of thirteen studies recruited both genders (male and female) [20, 22, 24, 26, 30, 37, 38, 42, 43], the participants in 2 were only females [40, 45], and 1 study recruited only men [25]. No information was provided about the participants’ gender in one study [36]. The mean age of participants in all 13 studies ranged from 23 to 76 years old [20, 22, 24–26, 30, 36–38, 40, 42, 43, 45].

Two studies investigated the effects of binaural beats in an experimental setup, inducing pain using painful heat laser pulses [20] or a surgical clamp on healthy volunteers [38]. In the 11 remaining studies, binaural beats intervention was used for patients undergoing surgery [22, 26, 30, 36, 37, 40, 42, 43] or a medical procedure including colonoscopy [24], mammography [45], and cystoscopy or ureteral stent removal [25].

#### Intervention features

The participants listened to binaural beats before the procedures in 3 studies for 5 min [45], 10 min [42], or 20 min [30], while in three other studies [24, 40, 43] listening to the binaural beats occurred during procedures, among which only one study reported the duration of the procedure (mean surgery time for the binaural beats and control groups were 80 and 88 min, respectively) [43]. In four studies listening to binaural beats started before the procedures and lasted until the end of the procedure [25, 26, 36, 37]. The exposure time before procedure was reported in three out of four studies (ranging from 10 to 30 min) [25, 26, 37]. Duration of procedure varied significantly between studies, lasting about 3 min for ureteral stent removal [25] to 170 min for lumbar spine surgical procedures [36]. The frequency of binaural beats has been specified in 7 studies that investigated the effects of binaural auditory beats on acute pain perception, from which two studies used a frequency of 4 Hz [24, 30], two used a frequency of 10 Hz [25, 42], and three studies used different frequencies within the theta to delta [43], delta to alpha [38] or alpha range [20]. Three out of six studies that did not report a specified frequency used hemispheric synchronization sounds for binaural beats intervention [22, 36, 37].

#### Measurements

Four out of thirteen studies measured the amount of analgesic consumption (fentanyl administration) [22, 36, 37] or sedative drug loading (dexmedetomidine loading dose) [43] as a measure of intraoperative nociception control. Six studies used a visual analog scale to measure pain scores immediately [42] and/or up to 24 h after the procedure [24–26, 37, 40]. Four studies used other non-specified numerical rating scales to evaluate pain intensity [20, 30, 38, 45].

### Risk of bias assessment

**Fig. 2:**
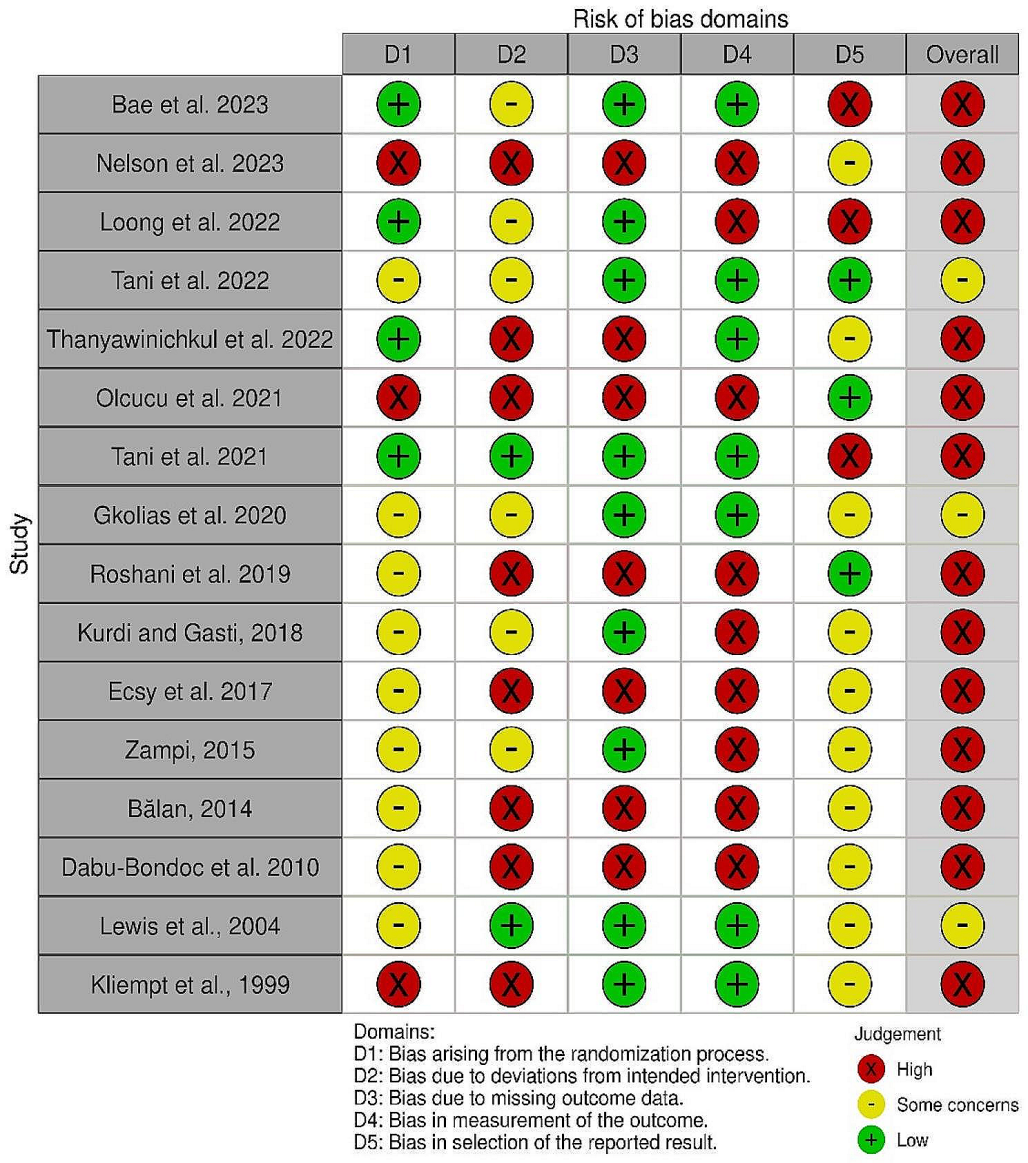
The results of risk of bias assessment for the included studies

**Fig. 3:**
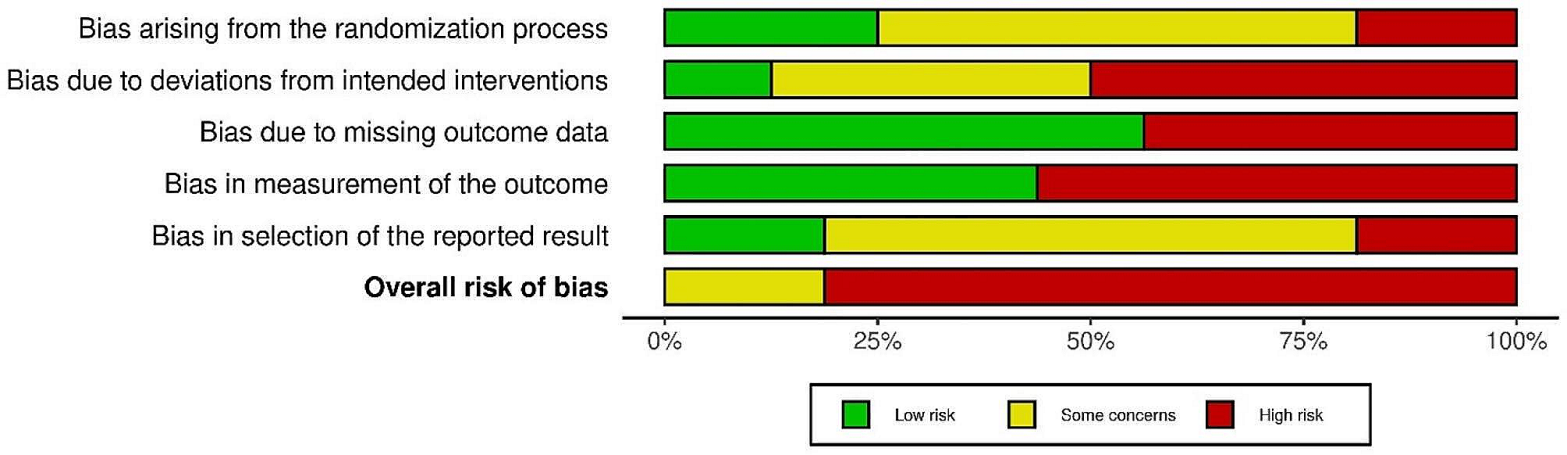
Overall risk of bias across studies

The results of the risk of bias assessment for the included studies based on the revised Cochrane risk-of-bias tool for randomized trials (RoB2) are illustrated in Figs. 2 and 3. The authors judged most RCTs (thirteen) to have a high risk of bias [20–22, 25, 26, 28, 30, 37, 38, 40, 42, 43, 45] mainly arising from the deviation from intended intervention, missing outcome data, and measurement of the outcome. Awareness of the outcome assessors about the intervention they received and the possible influence of this awareness on the assessment of the outcome was considered a highly potential source of bias. However, the high risk of bias in two studies was due to selective reporting [30, 43]. The remaining articles [3], being judged as showing some concerns [24, 27, 36], reported insufficient information about the randomization method or deviation from predesignated intervention, and/or lacked a prespecified protocol, which raised concerns about selection bias. No study was judged to have a low risk of bias.

### Quality of evidence assessment

The overall quality of evidence was low to very low mainly due to the risk of bias and varying effect sizes with wide confidence intervals (Table 2).

**Table 2:** GRADE evidence profile for binaural beats effects in acute and chronic pain

Outcomes		Quality assessments					Number of participants		Overall Quality of Evidence
	Number of studies	Risk of bias	Inconsistency	Indirectness	Imprecision	Publication bias	Binaural beats	Control	
Chronic pain perception	2 RCTs Gkolias,2020 Thanyawinichkul,2022	Serious^a^	Not serious^b^	Not serious^c^	Very serious^d^	Undetected^e^	20 10	20 12	⊕⊕⊖⊖ Low^f^
Acute pain perception	8 RCTs Dabu-Bondoc,2010 Kurdi,2018 Roshani,2019 Olcucu,2021 Tani,2021 Tani,2022 Loong,2022 Nelson,2023	Very serious^g^	Very serious^h^	Not serious^c^	Very serious^d^	Undetected^e^	20 59 30 102 20 42 31 20	20 62 30 127 20 48 30 20	⊕⊖⊖⊖ Very low^i^
Perioperative analgesic consumption	5 RCTs Kliempt,1999 Lewis,2004 Dabu-Bondoc,2010 Tani,2021 Bae,2023	Serious^a^	Serious^j^	Serious^k^	Very serious^d^	Undetected^e^	25 30 20 20 63	26 30 20 20 60	⊖⊖⊖⊖ Very low^i^

^a^ Two of the five risk of bias domains were judged as unclear or high in most studies

^b^ I-squared statistic (*I*^*2*^) < 50.0%

^c^ Population, interventions, and outcome measures were representative of our inclusion criteria

^d^ Wide confidence interval (CI) around the estimate of the effect (estimated by forest plots)

^e^ Based on Begg’s and Egger’s tests (*P* > 0.05)

^f^ The confidence in the effect estimate is limited (The true effect may be substantially different from the estimate of the effect)

^g^ More than two of the five risk of bias domains were judged as unclear or high in most studies

^h^ I-squared statistic (*I*^*2*^) > 75.0%

^i^ There is very little confidence in the effect estimate (The true effect is likely to be substantially different from the estimate of effect)

^j^ I-squared statistic 50.0%< (*I*^*2*^) < 75.0%

^k^ outcome measure was not representative of our inclusion criteria

## Discussion

The present study provides a comprehensive review of randomized controlled trials that investigated the efficacy of binaural auditory beats in acute and chronic pain management. Unlike previous reviews that did not assess the risk of bias [16, 29], the current systematic review assessed the risk of bias using the RoB-2 tool [32]. Sixteen randomized clinical trials were identified, among which three studies included patients with chronic pain [21, 27, 28] and thirteen assessed acute pain perception [20, 22, 24–26, 30, 36–38, 40, 42, 43, 45]. To facilitate the interpretation of findings, this review organized studies into three groups as follows: chronic pain perception, acute pain perception during experimental or clinical settings, and analgesic requirements.

Because of substantial heterogeneity with respect to the binaural beats exposure time and duration, the frequency of applied binaural beats, comparison group, and patients’ medical conditions so that they included patients undergone different surgeries or medical procedures a meta-analysis was inappropriate and quantitative results in each individual study are presented in forest plots.

### Effects of auditory binaural beats on chronic pain

**Fig. 4:**
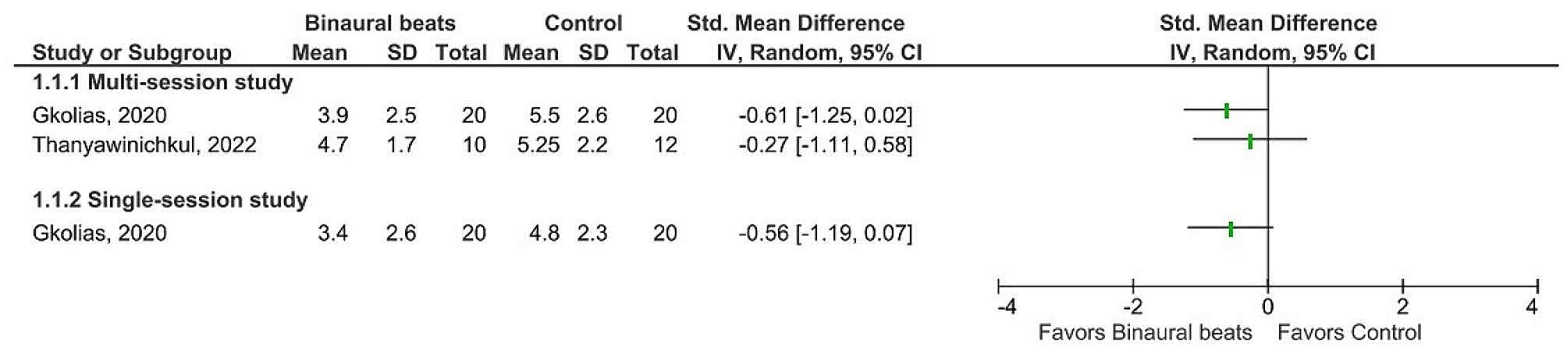
Efficacy of binaural beats intervention compared to control condition for change in chronic pain. Abbreviations: SD: Standard deviation; CI: Confidence interval

Three studies assessed the effects of binaural auditory beats intervention on chronic pain [21, 27, 28]. Although two studies reported a significantly lower pain score in the binaural beats group compared to the control group [21, 27], the forest plot showed no effect (Fig. 4). It needs to be noted that the effect size was estimated for just one of two studies that reported a significant between-group difference [27], because the other study did not provide sufficient data for estimating effect size [21]. The only study that assessed the effect of a single-session binaural beats intervention on pain perception reported that listening to binaural beats for 30 min was effective in reducing pain in patients suffering chronic pain with different etiologies, however, the forest plot showed no effect [27] (Fig. 4).

Although we did not find sufficient evidence for the efficacy of binaural beats in chronic pain in the available literature, the possible cause of the discrepancy between the results of published studies is briefly discussed in the following section.

The risk of bias was high in one out of two studies that showed the efficacy of binaural beats [21], while there were some concerns about the other study [27]. Also, the overall quality of evidence was low for the effect estimates in chronic pain. The risk of bias in the study that reported no between-group difference was also high principally due to deviation from intended intervention and missing outcome data [28].

Although Thanyawinichkul et al. [28] and Zampi [21] used an almost similar intervention protocol concerning the duration of binaural beats exposure and applied frequency, their studies differed in study design, participants’ pain origin, and sample size. Recruiting a small number of patients in a parallel randomized design in the study by Thanyawinichkul et al. may be the cause of failure to find a between-group difference compared to Zampi’s study that recruited a considerably higher number of patients in a crossover design. It is well known that a crossover design can yield a more efficient comparison between groups and balance covariates better in treatment and control arms because each person serves as his/her own control.

The other source of discrepancy may be the etiology of chronic pain, such that the two studies reporting pain reduction in favor of binaural beats intervention [21, 27] recruited participants with various types of chronic pain, while the patients in the study by Thanyawinichkul et al. suffered from chronic back pain [28]. In this regard, the literature has shown some differences in resting-state theta electroencephalography (EEG) power in patients who suffer from neuropathic pain or migraine headache, while in patients experiencing low back pain and fibromyalgia, no such changes have been reported [50]. It is important because baseline theta activity can predict pain reduction in response to some neuromodulatory pain treatments such as hypnosis [51]. Two of the three included studies [27, 28] evaluated theta activity in addition to pain perception. Decreased pain scores following theta binaural beat intervention in patients with various chronic pain in the study by Gkolias et al. were correlated with increased mean theta power [27]. However, in patients with low back pain, theta binaural beat neither induced significant pain relief nor caused changes in theta power [28]. These findings highlight the possible role of the etiology of chronic pain as a cause of differences between studies.

The only meta-analysis that considered binaural beats stimulation in chronic pain has reported the positive effects of theta entrainment in reducing chronic pain. It needs to be noted that this meta-analysis included only two studies both reporting significant pain reduction in binaural beats group [21, 27]. However, a new study reporting controversial results [28] challenges the previous findings. Furthermore, in the previous systematic reviews and meta-analyses, the quality of included studies has been assessed by a critical appraisal tool, while using risk-of-bias assessment tools is preferred for systematic reviews [52]. The risk of bias assessment in our study shows that the risk of bias was high in most included studies, which necessitates careful interpretation of the results.

In short, despite some reports regarding the influence of the short-term and multisession application of theta binaural beat on reducing pain perception in chronic pain, drawing a conclusion regarding the efficacy of binaural beats for this group of patients requires more high-quality studies.

### Effects of auditory binaural beats on acute pain

#### Effects of binaural beats on acute pain perception in experimental or clinical settings

Ten studies evaluated acute pain perception using a numerical rating scale in a clinical [24–26, 30, 37, 40, 42, 45] or an experimental setting [20, 38].

Listening to binaural auditory beats with a combination of tones within the delta to the alpha range or a pure alpha tone for 5 to 10 min has resulted in the perception of less pain induced by hemostat [38] or painful laser stimuli [20] in healthy subjects. However, the risk of bias in both studies was high mainly due to insufficient information regarding intervention deviations, missing data, and measurement of the outcomes.

**Fig. 5:**
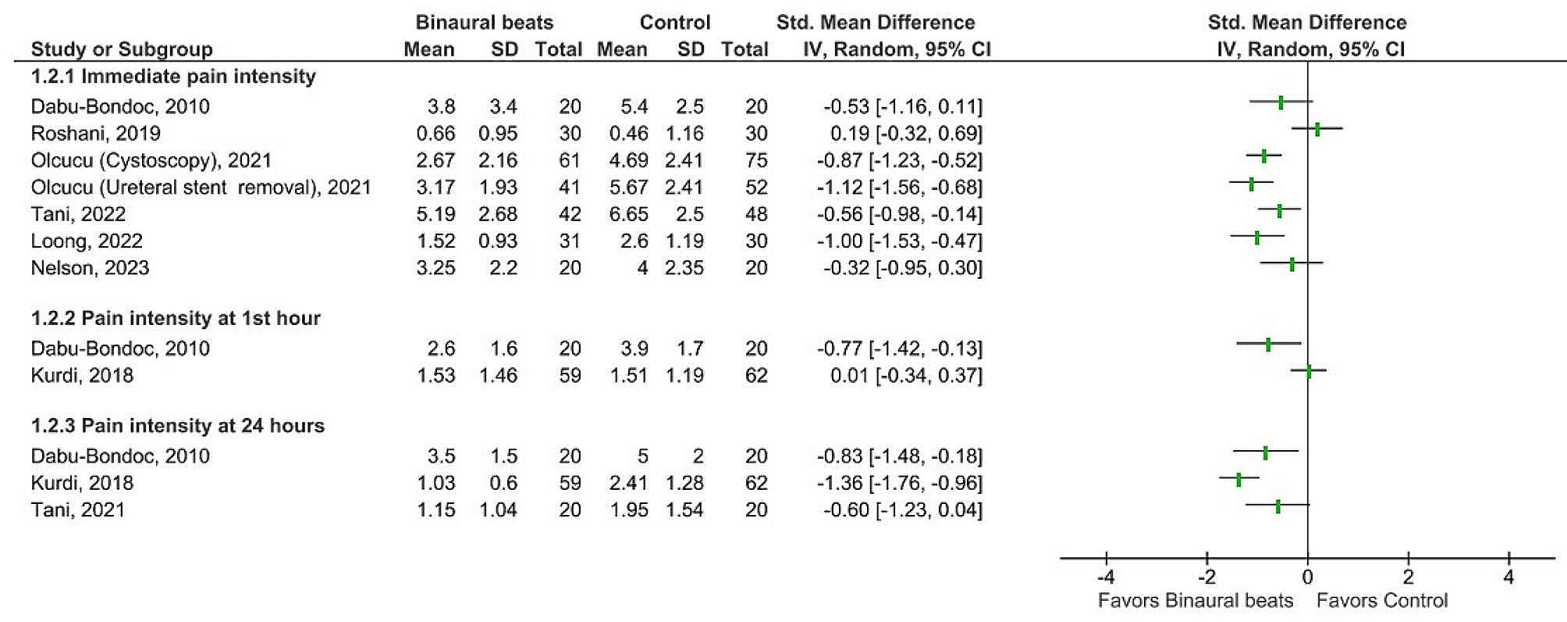
Efficacy of binaural beats intervention compared to control condition for change in acute pain scores of patients undergone medical procedures. Abbreviations: SD: Standard deviation; CI: Confidence interval

Eight studies assessed pain perception after medical procedures causing acute pain [24–26, 30, 37, 40, 42, 45], from which four studies with a high risk of bias [25, 37, 40, 42] and one with some concerns about bias [24] reported lower perceived pain (with medium to large effects) in the binaural beats group immediately or during the first day after the procedure (Fig. 5). However, two studies, both with high risks of bias, failed to show the advantage of binaural beats over acoustical stimulation or conventional treatment for patients who underwent total knee joint replacement [30] or eye surgery [26] (Fig. 5). However, the overall quality of evidence was rated as very low for acute pain perception.

Based on the results of the included studies, listening to alpha binaural beat at a frequency of 10 Hz for at least 10 min seems to be effective for reducing perceived pain immediately after phacoemulsification or cystoscopy and ureteral stent removal compared to no auditory stimulation [25, 42]. However, binaural auditory beat at a frequency of 4 Hz has been associated with different results. Patients who listened to theta binaural beat (4 Hz) before and during colonoscopy reported lower feelings of pain immediately after the procedure compared to those who listened to white noise [24], while for patients undergoing total knee joint replacement, no significant difference in pain scores was observed at different hours up to one day after surgery despite significantly lower morphine consumption after surgery in the binaural beats group [30]. Although these two studies used a binaural beat with the same frequency, other issues may have led to different findings. A meta-analysis that considered binaural beats intervention for anxiety, pain, and cognition found binaural beat masking as a potential factor influencing binaural beats efficacy [16]. Unmasked binaural beats are expected to result in larger effect sizes than those masked with music or white noise. In this regard, patients who underwent colonoscopy listened to a binaural beat that was masked with white noise [24] while those who underwent total knee replacement listened to a binaural beat associated with acoustic music in the background [30]. Differences between types of sound used for masking binaural beats might be a source of discrepancy. Another factor that needs consideration is the type of medical procedure. Colonoscopy without sedation and knee surgery under spinal anesthesia seems to be associated with different levels of anxiety and emotional and physical discomfort, which may have acted as mediators of the influence of binaural auditory beats on pain perception in these two distinct medical conditions. The delay in pain assessment after knee surgery compared to immediate reports collected after colonoscopy might be another source of discrepancy between the two studies.

Three remaining studies reporting acute pain perception after medical procedures that provided no information about the binaural beats frequency [26, 40, 45] also showed controversial results. Listening to binaural beats during cesarean section under spinal anesthesia resulted in significantly lower pain scores at 6 and 24 h but not 1 h after surgery compared to blank tape [40]. However, five minutes of exposure to binaural beats before mammography and listening to the binaural beats before and during eye surgery have not affected pain perception compared to conventional treatment [26, 45]. Comparing the studies in terms of the intervention setting excludes the exposure time or sample size as causes of discrepancy. However, not reporting the frequency of binaural beats used in these studies raises concerns about the applied frequency. Further, the high risk of bias for all three studies [26, 40, 45], especially the study by Nelson et al. with serious risks of bias in most domains [45], raises doubt about the validity of these findings.

Comparing the studies by Loong et al. and Roshani et al. conducted on patients under eye surgeries with a comparable sample size also highlights the possible role of binaural beats frequency in its effectiveness [26, 42]. Loong et al. used alpha binaural beat [42] while Roshani et al. did not report the frequency they used [26].

Altogether, listening to binaural auditory beats before or/ and during medical procedures seems to be effective for lowering acute pain perceived by the patients, and alpha binaural beat or a combination of tones in the range of delta to alpha seems to be more effective than theta frequency. However, the medical procedure may influence this efficacy.

#### Effects of binaural beats on analgesic requirements

**Fig. 6:**
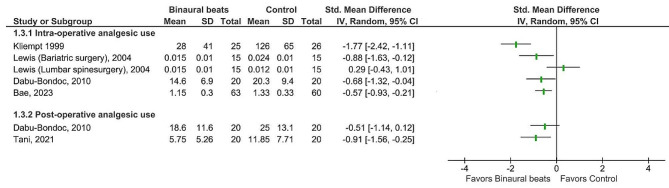
Efficacy of binaural beats intervention compared to control condition for change in perioperative analgesic requirements of patients undergone surgery. Abbreviations: SD: Standard deviation; CI: Confidence interval

Four studies assessed intraoperative analgesic consumption and all reported a reduction in analgesic requirements in the binaural beats intervention group compared to blank tape with medium to large effect sizes [22, 36, 37, 43] (Fig. 6). Among these studies, the risk of bias was high in three [22, 37, 43], and there were some concerns about one [36]. Two studies reported decreased fentanyl consumption during general surgeries requiring anesthesia in binaural beats groups [22, 37], one showed lower dexmedetomidine loading dose in the binaural beats group during orthopedic surgeries [43], while one study showed a significant decrease in required fentanyl in the binaural beats group only in patients undergoing bariatric surgical procedures and not those undergoing lumbar spine surgical procedures [36]. As the study setting was the same for the two groups of patients, the differences in the results might be due to the type of surgery. The results show that the patients who underwent bariatric surgery in the control group required about double doses of fentanyl during surgery compared with patients who underwent lumbar surgeries. Requiring this high dose of the analgesic drug suggests some differences between the two patient groups at baseline. Obese patients usually represent high levels of anxiety and depression [53], while about 20% of orthopedic patients have shown anxiety levels possibly warranting treatment [54]. Anxiety and depression have been suggested as factors influencing postoperative pain perception in all clinical settings [55], including bariatric surgery [56]. Previous studies have reported the effectiveness of binaural beats on anxiety reduction [57]. Therefore, considering applying the same intervention method for two different groups of patients in the study by Lewis et al., the efficacy of binaural beats on the analgesic requirements in patients who underwent bariatric surgery might be mediated by the anxiolytic effects of binaural beats [36].

Also, there was a significant effect of binaural beats (with a large effect size) (Fig. 6) on patient-controlled postoperative morphine consumption after total knee replacement [30]. Although, no significant effect of binaural beats on analgesic requirements in the post-anesthesia care unit was observed for those who underwent different surgeries in the study of Dabu-Bondoc et al., patients reported a significantly less perceived pain at 1 and 24 h after surgery [37]. This discrepancy may be due to earlier discharge of patients in binaural beats group compared to controls in the study by Dabu-Bondoc et al. [37].

Altogether, the current literature with very low quality of evidence suggests the effectiveness of binaural beats in reducing perioperative analgesic requirements, however, the medical condition seems to be a contributing factor to this efficacy.

### Strengths and limitations

We comprehensively searched five databases to assess the effectiveness of binaural auditory beats in acute and chronic pain. This is the first review in this field that has evaluated the risk of bias using the revised Cochrane risk-of-bias tool for randomized trials (RoB2). Risk of bias assessment is preferred to critical appraisal in systematic reviews. However, there are some limitations that must be noted. Due to the heterogeneity of the included studies in terms of acute or chronic pain, medical procedures, and binaural beat frequency, a meta-analysis was not performed, but forest plots were provided to represent effect size for individual papers and a comprehensive narrative review was performed. Further, the risk of bias was high in most included studies which could limit the evidence-based conclusions and necessitate high caution when interpreting the findings. The small number of studies, especially in chronic pain, and the results of risk of bias assessment necessitate further high-quality studies with sufficiently large sample sizes and homogenous participants to evaluate the efficacy of binaural beats for chronic pain control.

### Implication for the future

According to our results, although binaural auditory beats intervention seems to be effective for pain relief in acute condition and shows some potential for pain reduction in chronic pain, its effectiveness may be dependent on some factors such as the patient’s medical condition and the frequency of binaural beats. Since pain perception is highly subjective and most included studies used self-reported numerical rating scales for assessing pain after the intervention, high-quality double-blind randomized clinical trials providing sufficient information about the randomization, concealment, blindness, and the assessment of outcome as a major source of bias is recommended. The exposure moment and duration as well as the frequency of binaural beats need to be precisely considered in future studies. Comparing binaural beats at delta, theta, and alpha frequencies in the same study population could expand our knowledge about the most effective frequency for pain relief.

## Conclusion

Based on the available literature, it seems that pain with various origins may be influenced differently by binaural auditory beats. However, our systematic review identifies binaural auditory beats as a potential non-pharmacological intervention for reducing pain, especially in acute pain in different medical conditions. Nevertheless, the authors prefer to be watchful about making an absolute conclusion due to the low to very low quality of evidence and high risk of bias identified across the included studies. So, future studies recruiting homogenous populations are suggested for drawing a more reliable conclusion regarding the efficacy of binaural auditory beats in reducing pain perception in acute and chronic pain.

### Electronic supplementary material

Below is the link to the electronic supplementary material.

**Supplementary Material 1:** PRISMA 2020 Checklist

**Supplementary Material 2:** Detail of search strategy in all databases included in this review

## Data Availability

All data generated or analyzed during this study are included in this published article.

## Abbreviations

EEG: Electroencephalography
MPI: Multidimensional Pain Inventory
PRISMA: Preferred Reporting Items for Systematic Reviews and Meta-Analyses
PROSPERO: Prospective Register of Systematic Reviews
RoB2: Risk-of-Bias 2 Assessment Tool
