# Supplementary material for: Does brain entrainment using binaural auditory beats affect pain perception in acute and chronic pain?: a systematic review
Source: BMC Complement Med Ther. 2024 Jan 12;24:34. doi: 10.1186/s12906-024-04339-y (PMC10785528; doi:10.1186/s12906-024-04339-y)
Supplement: Supplementary file 2 — Supplementary Material 2: Detail of search strategy in all databases included in this review [file 12906_2024_4339_MOESM2_ESM.pdf]

**Detail of search strategy in all databases included in this review**

**Pubmed**

((((((((((pain[Title/Abstract]) OR (pain[MeSH Terms])) OR (ache[Title/Abstract])) OR (ache[MeSH Terms])) OR (analgesia[Title/Abstract])) OR (analgesia[MeSH Terms])) AND (binaural beat[Title/Abstract])) OR (binaural beats[Title/Abstract])) OR (binaural auditory beat[Title/Abstract])) OR (binaural beat entrainment[Title/Abstract])) OR (hemispheric synchronization[Title/Abstract])

**Scopus**

( ( TITLE-ABS-KEY ( binaural AND beat ) OR TITLE-ABS-KEY ( binaural AND beats ) OR TITLE-ABS-KEY ( binaural AND auditory AND beats ) OR TITLE-ABS-KEY ( binaural AND beat AND entrainment ) OR TITLE-ABS-KEY ( hemispheric AND synchronization ) ) ) AND ( ( TITLE-ABS-KEY ( pain ) OR TITLE-ABS-KEY ( ache ) OR TITLE-ABS-KEY ( analgesia ) ) )

**Embase**

#1 'pain'/exp OR pain

#2 'analgesia'/exp OR analgesia

#3 ache

#4 'binaural beat'/exp OR 'binaural beat' OR (binaural AND beat)

#5 binaural beats OR (binaural AND beats)

#6 'binaural auditory beat' OR (binaural AND auditory AND beat)

#7 'binaural beat entrainment' OR (binaural AND beat AND ('entrainment'/exp OR entrainment))

#8 'hemispheric synchronization' OR (hemispheric AND ('synchronization'/exp OR synchronization)))

# 9 (#1 OR #2 OR #3)

#10 (#4 OR #5 OR #6 OR #7 OR #8)

#10 (#9 AND #10)

**Cochrane**

#1 (pain):ti,ab,kw OR (ache):ti,ab,kw OR (analgesia):ti,ab,kw (Word variations have been searched)

#2 MeSH descriptor: [Pain] explode all trees

#3 MeSH descriptor: [Analgesia] explode all trees

#4 (binaural beat):ti,ab,kw OR (binaural beats):ti,ab,kw OR (binaural auditory beat):ti,ab,kw OR (binaural beat entrainment):ti,ab,kw OR (hemispheric synchronization):ti,ab,kw (Word variations have been searched)

#5 #1 OR #2 OR #3

#6 #4 AND #5

**WOS**

#1 ((TS=(pain)) OR TS=(ache)) OR TS=(analgesia)

# 2 ((((TS=(binaural beat)) OR TS=(binaural beats)) OR TS=(binaural auditory beat)) OR TS=(binaural beat entrainment)) OR TS=(hemispheric synchronization)

#3 (# 1 AND # 2)
